# Retraction: Fabrication of hollow CoS_1.097_ prisms toward supercapactior performance

**DOI:** 10.1039/c9ra90056j

**Published:** 2019-07-16

**Authors:** Ruili Zhang, Yuntao Yang, Ping Yang

**Affiliations:** School of Material Science and Engineering, University of Jinan Jinan 250022 P. R. China mse_yangp@ujn.edu.cn

## Abstract

Retraction of ‘Fabrication of hollow CoS_1.097_ prisms toward supercapactior performance’ by Ruili Zhang *et al.*, *RSC Adv.*, 2019, **9**, 10814–10819.

We, the named authors, hereby wholly retract this *RSC Advances* article due to extensive overlap with the text, data and figures published in ref. 1, which means that this *RSC Advances* article is redundant. All the figures and tables in this *RSC Advances* article have been reproduced from ref. 1.

The authors would like to apologise for any inconvenience to readers.

Signed: Ruili Zhang and Ping Yang.

Date: 8^th^ July 2019.

We do not have current contact details for the second author of the article, Yuntao Yang, as he has graduated and therefore has not been able to comment on the retraction.

Retraction endorsed by Andrew Shore, Executive Editor, *RSC Advances*.

## Supplementary Material
